# Respiratory Virus Activity — United States, July 1, 2024–June 30, 2025

**DOI:** 10.15585/mmwr.mm7506a2

**Published:** 2026-02-19

**Authors:** Benjamin J. Silk, Mila M. Prill, Amber K. Winn, Monica E. Patton, Heidi L. Moline, Michael Melgar, Kevin C. Ma, Clinton R. Paden, Lydia J. Atherton, Diba Khan, Christopher A. Taylor, Ayzsa Tannis, Kadam Patel, Leah A. Goldstein, Krista Kniss, Angiezel Merced-Morales, Natalie Thornburg, Fatimah S. Dawood

**Affiliations:** ^1^Coronavirus and Other Respiratory Viruses Division, National Center for Immunization and Respiratory Diseases, CDC; ^2^Eagle Health Analytics, LLC, San Antonio, Texas; ^3^Alutiiq, LLC, Chesapeake, Virginia; ^4^Influenza Division, National Center for Immunization and Respiratory Diseases, CDC.

**Summary**

**What is already known about this topic?**

CDC monitors the activity and severity of respiratory viruses using data from complementary surveillance systems. Weekly data are published online.

**What is added by this report?**

Severe outcomes from COVID-19 and respiratory syncytial virus (RSV) continue to occur, especially among young children and older adults. COVID-19 was associated with an estimated 290,000–450,000 hospitalizations and 34,000–53,000 deaths; RSV was associated with 190,000–350,000 hospitalizations and 10,000–23,000 deaths. All sequenced circulating U.S. SARS-CoV-2 viruses remained descendants of the JN.1 variant, representing the first season without a SARS-CoV-2 strain replacement since the beginning of the COVID-19 pandemic.

**What are the implications for public health practice?**

Respiratory virus surveillance remains critical for preparedness and prevention monitoring. Staying up to date with recommended respiratory virus vaccinations can protect against severe COVID-19, RSV, and influenza.

## Abstract

Respiratory viruses are common causes of upper and lower respiratory tract illness and can also result in hospitalization and death. CDC conducts national surveillance using multiple systems to monitor ongoing and seasonal changes in the activity of selected respiratory viruses. This report summarizes U.S. trends in endemic respiratory virus activity during July 2024–June 2025. For SARS-CoV-2 and respiratory syncytial virus (RSV), national and regional trends; population-based hospitalization rates; vital records death counts; and preliminary estimates of associated illnesses, outpatient visits, hospitalizations, and deaths are described, as well as genetic characterization of circulating SARS-CoV-2 viruses. Some viruses, including SARS-CoV-2, showed bimodal peaks in positive laboratory test results, whereas others, including RSV and influenza viruses, were characterized by a single peak. The highest COVID-19–associated hospitalization rates were reported among adults aged ≥75 years (932.6 per 100,000 persons), infants aged <6 months (285.6), and adults aged 65–74 years (274.4). RSV-associated hospitalization rates were highest among infants aged <12 months (1,116.7 per 100,000; 95% CI = 1,078.4–1,157.9), children aged 12–23 months (770.6; 95% CI = 743.1–800.3), and adults aged ≥75 years (426.9; 95% CI = 366.6–510.8). COVID-19 was associated with an estimated 290,000–450,000 hospitalizations and 34,000–53,000 deaths; RSV was associated with 190,000–350,000 hospitalizations and 10,000–23,000 deaths. All circulating SARS-CoV-2 lineages were Omicron JN.1 descendants. Staying up to date with recommended COVID-19, RSV, and influenza vaccinations remains important to reducing the risk for severe disease caused by these viruses.

## Introduction

The circulation of many respiratory viruses, which are common causes of upper and lower respiratory tract illnesses, varies seasonally. Although respiratory viral infections usually cause mild or moderate illnesses, such as the common cold, lower respiratory tract infections can result in hospitalization and death. CDC uses multiple surveillance systems to systematically monitor selected respiratory virus activity in the United States, including SARS-CoV-2 (the virus that causes COVID-19), influenza viruses, respiratory syncytial virus (RSV), human metapneumovirus (hMPV), rhinoviruses and enteroviruses (RV/EV), parainfluenza viruses (PIV) types 1–4, common human coronaviruses (229E, NL63, OC43, and HKU1), and respiratory adenoviruses. This report summarizes U.S. trends in endemic respiratory virus activity during July 2024–June 2025; hospitalization rates, vital records death counts, and estimates of illnesses, outpatient visits, hospitalizations, and deaths for COVID-19 and RSV; and genomic surveillance data for SARS-CoV-2. CDC publishes a separate summary of influenza activity, morbidity, and mortality each season. Ongoing national respiratory virus surveillance is critical for characterizing seasonality, providing information to guide health system preparedness and the timing of prevention product use for certain respiratory viruses, and identifying threats caused by new strains or novel viruses.

## Methods

### Data Sources

Data from six national surveillance systems (Box) were analyzed for this report. Data on COVID-19 and RSV vaccinations status and COVID-19 antiviral treatment are not systematically collected by some systems and were not analyzed for this report. All findings were limited to data reported during July 1, 2024–June 30, 2025, unless otherwise specified. This activity was reviewed by CDC, deemed not research, and was conducted consistent with applicable federal law and CDC policy.*

BOX **Surveillance systems used in analyses — United States, July 2024–June 2025**

**1. National Respiratory and Enteric Virus Surveillance System (NREVSS)**

- **Data source**: specimen testing results from children and adults, all ages
- **Settings:** participating clinical laboratories, commercial reference laboratories, and public health laboratories (influenza data are limited to clinical laboratory testing data)
- **Inclusion dates**: June 30, 2024–June 28, 2025
- **Type of surveillance**: passive (voluntary) reporting of laboratory test results as weekly aggregate counts
- **Reporting sources included**: approximately 345 participating laboratories representing all 50 states and the District of Columbia. Data were included from laboratories consistently reporting SARS-CoV-2 tests (n = 298 laboratories), respiratory syncytial virus (RSV) tests (273), rhinoviruses/enteroviruses tests (156), respiratory adenovirus tests (149), parainfluenza virus tests (222), common human coronaviruses tests (125) and human metapneumovirus tests (190). Current participating laboratories are shown here: Participating Labs | NREVSS | CDC
- **RSV season onset and offset:** onset and offset were identified, respectively, as the week ending date corresponding to the first and last of 2 consecutive weeks when the percentage of weekly tests positive for RSV reached or exceeded 3%

**2. COVID-19–Associated Hospitalization Surveillance Network (COVID-NET)**

- **Population:** all ages (children and adults) hospitalized with laboratory-confirmed SARS-CoV-2 infection identified through provider-driven testing
- **Settings:** inpatient
- **Inclusion dates**: July 1, 2024–June 30, 2025
- **Type of surveillance:** active, population-based sentinel surveillance network
- **Catchment area included**: residents receiving care in approximately 300 participating hospitals in select counties in California, Colorado, Connecticut, Georgia, Maryland, Michigan, Minnesota, New Mexico, New York, North Carolina, Oregon, Tennessee, and Utah
- **Unadjusted rate calculation:** COVID-19–associated hospitalizations are defined as those among persons who have received a positive SARS-CoV-2 reverse transcription–polymerase chain reaction (RT-PCR) or rapid antigen detection test result during hospitalization or ≤14 days before admission. Unadjusted COVID-NET hospitalization rates (number of hospitalizations per 100,000 population) were estimated by dividing total catchment-area COVID-19 hospitalizations by U.S. Census Bureau vintage unbridged-race postcensal population estimates for the counties or county equivalents in the surveillance catchment area. COVID-NET conducts population-based surveillance in which every hospitalization meeting the case definition is ascertained. Because hospitalization rates reflect actual measured rates in the catchment area population, 95% CIs are not calculated

**3. New Vaccine Surveillance Network (NVSN)**

- **Population:** children and adolescents <18 years
- **Settings:** outpatient (outpatient clinics, urgent care clinics, and emergency departments); inpatient
- **Inclusion dates:** July 1, 2024–June 30, 2025
- **Type of surveillance:** active, prospective, population-based sentinel surveillance network for acute respiratory illness (ARI)
- **Medical centers included:** Vanderbilt University Medical Center (Tennessee), University of Rochester Medical Center (New York), Cincinnati Children’s Hospital Medical Center (Ohio), Texas Children’s Hospital (Texas), Seattle Children’s Hospital (Washington), Children’s Mercy Hospital (Missouri), and University of Pittsburgh Medical Center Children’s Hospital of Pittsburgh (Pennsylvania)
- **Adjusted rate calculation:** All children hospitalized with ARI (based on a standard case definition) have respiratory samples collected (or residual clinical samples retained) and tested for respiratory viruses by RT-PCR. NVSN population-based numerators are adjusted using multipliers for the observed number of enrolled hospitalizations to account for weeks with <7 days of surveillance, the percentage of eligible children not enrolled, sensitivity of RSV RT-PCR testing (87.6%) for RSV rates only, and the market share of each enrollment hospital site for the estimated proportion of ARI hospitalizations ascertained in the catchment area. Adjusted rates were estimated per 100,000 children, and 95% CIs were determined to account for multiplier uncertainty by percentiles based on 1,000 bootstrap samples for each rate

**4. RSV-Associated Hospitalization Surveillance Network (RSV-NET)**

- **Population:** all ages (children and adults) hospitalized with laboratory-confirmed RSV infection identified through provider-driven testing
- **Settings:** inpatient
- **Inclusion dates:** July 1, 2024–June 30, 2025
- **Type of surveillance:** active, population-based sentinel surveillance network
- **Catchment area included:** residents receiving care in approximately 300 participating hospitals in select counties in California, Colorado, Connecticut, Georgia, Maryland, Michigan, Minnesota, New Mexico, New York, North Carolina, Oregon, Tennessee, and Utah
- **Adjusted rate calculation:** RSV-associated hospitalizations are defined as those among persons who have received a positive RT-PCR or rapid antigen detection test result during hospitalization or ≤14 days before admission. Unadjusted RSV-NET hospitalization rates (number of hospitalizations per 100,000 population) were estimated by dividing total catchment-area RSV-associated hospitalizations by U.S. Census Bureau vintage unbridged-race postcensal population estimates for the counties or county equivalents in the surveillance catchment area. RSV-NET rates are adjusted with multipliers to account for underdetection due to diagnostic test sensitivity and testing practices. Adjusted rates are presented with 95% CIs to account for multiplier uncertainty

**5. National Vital Statistics System (NVSS)**

- **Data source:** provisional mortality data on the CDC Wonder Online Database
- **Population:** all ages (children and adults)
- **Settings:** any setting within the United States
- **Inclusion dates:** July 1, 2024–June 30, 2025
- **Type of surveillance:** passive, population-based reporting of death certificate data
- **Catchment area:** compiled from data provided by the 57 vital statistics jurisdictions through the Vital Statistics Cooperative Program
- **Mortality ascertainment**: includes all deaths among U.S. residents occurring within the United States for which COVID-19 or RSV was listed on the death certificate as the underlying (primary) or contributing cause of death in the chain of events leading to death. COVID-19–associated deaths were identified with an *International Classification of Diseases, Tenth Revision* (ICD-10) code of U07.1 (COVID-19) assigned as a cause of death. RSV-associated deaths were identified with ICD-10 code J12.1 (respiratory syncytial virus pneumonia), J20.5 (acute bronchitis due to respiratory syncytial virus), or J21.0 (acute bronchiolitis due to respiratory syncytial virus)

**6. SARS-CoV-2 National Genomic Surveillance and RSV Subtyping**

- **Data source:** specimens and sequencing or subtyping results from children and adults, all ages
- **Settings:** For SARS-CoV-2: National SARS-CoV-2 Strain Surveillance (NS3) program, public sequence data repositories, and before June 2025, CDC-contracted commercial laboratories. Sequences from public repositories are limited to those meeting baseline surveillance criteria, which ensures that they are representative. For RSV: surveillance specimens received from NVSN and state and local public health laboratories
- **Inclusion dates:** June 8, 2024**–**July 5, 2025
- **Type of surveillance:** passive, laboratory-based reporting
- **Jurisdictions included:** 56 U.S. jurisdictions for SARS-CoV-2 (50 U.S. states, District of Columbia, American Samoa, Guam, Northern Mariana Islands, Puerto Rico, and U.S. Virgin Islands) and 15 U.S. jurisdictions for RSV (Arkansas, California, Louisiana, Missouri, Nevada, New York, North Carolina, Ohio, Pennsylvania, South Carolina, Tennessee, Virginia, Washington, Wisconsin, and Wyoming)

### Data Analysis

**Respiratory virus activity**. The National Respiratory and Enteric Virus Surveillance System (NREVSS) includes results from clinical testing of persons seeking medical care reported by clinical, reference, and public health laboratories throughout the United States. For this report, influenza data were limited to submissions from clinical laboratories and influenza-collaborating laboratories to monitor the timing and intensity of influenza activity. Data from NREVSS were analyzed to provide virus-specific information on percentages of laboratory tests that were positive (percent positive).

**Hospitalizations and deaths associated with COVID-19 and RSV.** Rates of laboratory-confirmed hospitalization associated with COVID-19 and RSV are monitored by three population-based surveillance systems: 1) the COVID-19–Associated Hospitalization Surveillance Network (COVID-NET), 2) the RSV-Associated Hospitalization Surveillance Network (RSV-NET), and 3) the New Vaccine Surveillance Network (NVSN). COVID-NET and RSV-NET conduct surveillance among residents of all ages in predefined catchment areas to calculate hospitalization rates overall and by age. NVSN conducts surveillance among children and adolescents aged <18 years, with systematic testing of patients with acute respiratory illness at seven U.S. medical centers. Counts of deaths associated with COVID-19 and RSV are described using provisional death certificate data compiled by the National Vital Statistics System (NVSS) based on codes for specific underlying or contributing causes of death.

**Modeling estimates.** Mathematical models using previously described methods were used to estimate COVID-19 and RSV illnesses, outpatient visits, hospitalizations, and deaths (*1*). These estimates characterize the impact of these infections on the U.S. population.

**SARS-CoV-2 genomic surveillance and RSV subtyping.** CDC tracks the genomic evolution of SARS-CoV-2 and estimates variant proportions by integrating sequence data from the National SARS-CoV-2 Strain Surveillance program, public sequence data repositories and, before June 2025, CDC-contracted commercial laboratories. CDC identifies RSV subtype from surveillance specimens received from NVSN and state and local public health laboratories. Data were analyzed using SAS (version 9.4; SAS Institute).

## Results

### Respiratory Virus Activity

**FIGURE 1:**
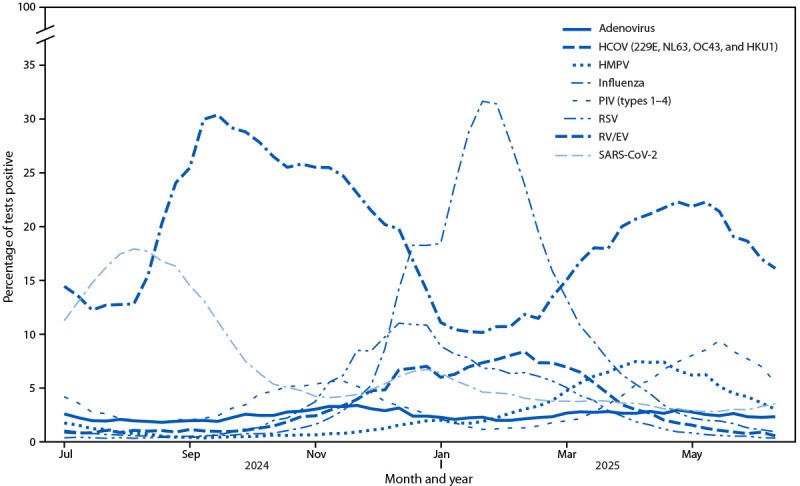
Weekly percentage of laboratory test results positive for respiratory viruses* — National Respiratory and Enteric Virus Surveillance System, United States, June 30, 2024–June 28, 2025^†^ **Abbreviations:** HCOV = human coronavirus; HMPV = human metapneumovirus; PIV = parainfluenza viruses; RSV = respiratory syncytial virus; RV/EV = rhinovirus/enterovirus. * Influenza data are limited to clinical laboratory testing data. ^†^ As of December 5, 2025.

During June 30, 2024–June 28, 2025, bimodal peaks occurred in the percentage of specimens that tested positive for SARS-CoV-2, RV/EV, and PIV 1–4 at laboratories participating in NREVSS (Figure 1). In contrast, single peaks occurred in the percentage of positive laboratory results for RSV, influenza viruses, hMPV, and common coronaviruses.^†^

### SARS-CoV-2

During the reporting period, 297 laboratories reported 3,961,594 SARS-CoV-2 polymerase chain reaction test results, 260,883 (6.6%) of which were positive. Nationally, the highest percentage of positive SARS-CoV-2 test results (i.e., the peak) (17.9%) occurred during the week ending August 10, 2024 (week 32), and the lowest percentage of positive tests (the trough) (4.0%) occurred during the week ending November 16, 2024 (week 46). A second peak (6.7%) occurred during the week ending January 4, 2025 (week 1) followed by a trough (2.7%) during the week ending May 24, 2025 (week 21). By U.S. Department of Health and Human Services (HHS) region, the peaks within the first elevated period ranged from weeks 29 (HHS Region 9) to 35 (HHS Regions 3 and 5) and within the second elevated period from weeks 48 (HHS Region 6) to 11 (HHS Region 4) (Supplementary Table 1).

### Rhinovirus/Enterovirus and Parainfluenza

The two peaks in the percentage of positive RV/EV test results occurred in the fall (30.4%) during the week ending September 21, 2024 (week 38) and the spring (22.3%) during the week ending May 10, 2025 (week 19) (Figure 1). The weekly percentage of positive RV/EV test results ranged from 10.1% to 30.4%. The two peaks in the percentage of positive PIV 1–4 test results occurred in the winter (5.6%) during the week ending November 23, 2024 (week 47) and spring (9.4%) during the week ending May 31, 2025 (week 22).

### Respiratory Syncytial Virus

A total of 3,767,034 RSV specimen tests were reported; among these, 178,911 (4.8%) results were positive. The percentage of positive test results peaked at 11.0% during the week ending December 21, 2024 (week 51). Nationally, RSV epidemic onset^§^ occurred during the week ending November 9, 2024 (week 45), and epidemic offset occurred during the week ending March 29, 2025 (week 13). Within the continental United States, the earliest RSV epidemic onset occurred in Florida (week 39, ending September 28, 2024), followed by the South (HHS Region 6, week ending October 5, 2024 [week 40]) and the Southeast (HHS Region 4, week ending October 19, 2024 [week 42]); the peak percentage of positive RSV test results also varied by region(Supplementary Table 2).^¶^

### Influenza, Human Metapneumovirus, and Common Coronavirus

The percentage of positive influenza virus test results from clinical laboratories peaked (31.6%) during the week ending February 1, 2025 (week 5) (Figure 1). The peak in percentage of positive hMPV test results (7.4%) occurred during the week ending April 19, 2025 (week 16). The peak in percentage of positive common coronavirus (types 229E, NL63, OC43, and HKU1) test results (8.3%) occurred during the week ending February 22, 2025 (week 8). A pattern of elevated respiratory adenovirus activity was not observed in the 2024–25 season.

### Hospitalizations and Deaths

**FIGURE 2:**
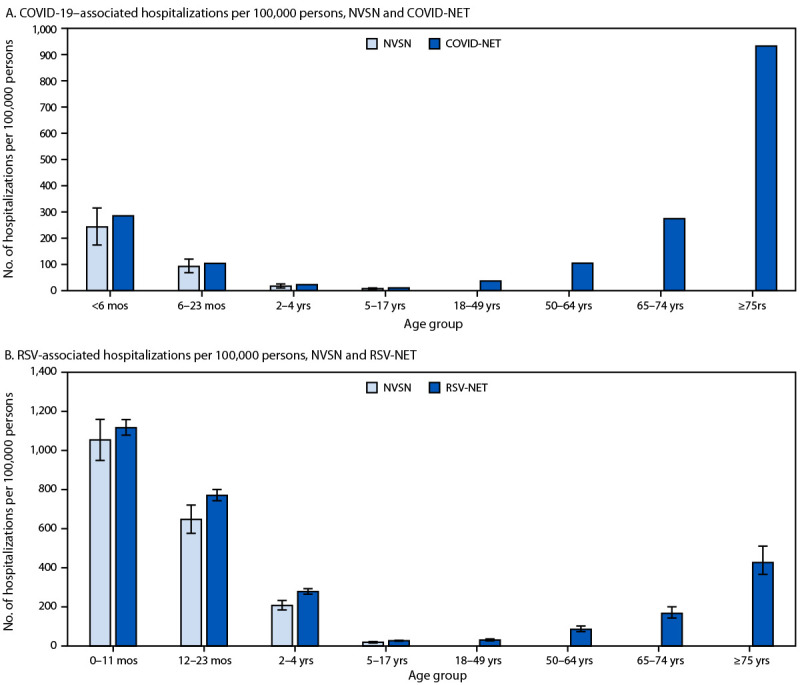
Hospitalization rates* associated with COVID-19^†^^,^^§^ (A) and respiratory syncytial virus^¶^ (B), by age group — United States, July 1, 2024–June 30, 2025**^,††^ **Abbreviations:** COVID-NET = COVID-19 Hospitalization Surveillance Network; NVSN = New Vaccine Surveillance Network; RSV = respiratory syncytial virus; RSV-NET = Respiratory Syncytial Virus Hospitalization Surveillance Network. * With 95% CIs indicated by error bars. **^†^** COVID-NET conducts population-based surveillance in which every hospitalization meeting the case definition is ascertained. Because hospitalization rates reflect actual measured rates in the catchment area population, 95% CIs are not calculated. ^§^ NVSN surveillance is pediatric only. Population-based numerators are adjusted using multipliers for the observed number of enrolled hospitalizations to account for weeks with <7 days of surveillance, the percentage of eligible children not enrolled, sensitivity of RSV reverse transcription–polymerase chain reaction testing (87.6%) for RSV rates only, and the market share of each enrollment hospital site for the estimated proportion of acute respiratory illness hospitalizations ascertained in the catchment area. Adjusted rates were estimated per 100,000 children, and 95% CIs were determined to account for multiplier uncertainty by percentiles based on 1,000 bootstrap samples for each rate. ^¶^ RSV-NET rates are adjusted with multipliers to account for underdetection due to diagnostic test sensitivity and testing practices. Adjusted rates are presented with 95% CIs to account for multiplier uncertainty. ** Age groups for hospitalized children aged <2 years are <6 months and 6–23 months for COVID-19 and 0–11 months and 12–23 months for RSV. ^††^ As of December 19, 2025.

**COVID-19.** Based on analysis of COVID-NET data, adults aged ≥75 years experienced the highest rate of COVID-19–associated hospitalization (number of hospitalizations per 100,000 persons) (932.6), followed by infants aged <6 months (285.6), adults aged 65–74 years (274.4) and 50–64 years (104.7), and children aged 6–23 months (104.1) (Figure 2). Pediatric COVID-19–associated hospitalization rates estimated by NVSN were highest among infants aged <6 months (243.0; 95% CI = 174.1–315.2), followed by children aged 6–23 months (92.5; 95% CI = 68.6–120.6).

A total of 34,981 COVID-19–associated deaths were recorded by NVSS, including 187 (0.5%) among children and adolescents aged <18 years, 3,781 (10.8%) among persons aged 18–64 years, and 30,923 (88.4%) among adults aged ≥65 years. During October 1, 2024–July 5, 2025, COVID-19 was associated with an estimated 10.4–16.7 million illnesses, 2.5–4 million outpatient visits, 290,000–450,000 hospitalizations, and 34,000–53,000 deaths.

**RSV.** RSV-associated hospitalization rates estimated by RSV-NET were highest among infants aged 0–11 months (1,116.7; 95% CI = 1,078.4–1,157.9), followed by children aged 12–23 months (770.6; 95% CI = 743.1–800.3) and adults aged ≥75 years (426.9; 95% CI = 366.6–510.8). Pediatric RSV-associated hospitalization rates estimated by NVSN were highest among infants aged 0–11 months (1,053.9; 95% CI = 949.0–1,159.0), followed by children aged 12–23 months (647.5; 95% CI = 576.7–720.8).

Among 672 RSV-associated deaths recorded by NVSS, 31 (4.6%) occurred among children and adolescents aged <18 years, 89 (13.2%) among adults aged 18–64 years, and 552 (82.1%) among adults aged ≥65 years. During October 1, 2024–May 3, 2025, RSV was associated with an estimated 3.6–6.5 million outpatient visits, 190,000–350,000 hospitalizations, and 10,000–23,000 deaths. 

### SARS-CoV-2 Lineages and RSV Subtypes

A total of 92,703 SARS-CoV-2 sequences from 56 U.S. jurisdictions were analyzed. Among these, 2% were from the National SARS-CoV-2 Strain Surveillance program, 22% were from commercial laboratories, and 76% were tagged baseline sequences from public repositories. All SARS-CoV-2 lineages circulating at ≥1% prevalence during this period were Omicron JN.1 descendant lineages. During at least one 4-week period, KP.3.1.1-like and LP.8.1 lineages accounted for >50% of sequenced viruses, and KP.2-like, KP.3, LB.1-like, XEC, and XFG lineages accounted for >20% of sequenced viruses (Supplementary Figure). Among 630 RSV specimens from 16 U.S. jurisdictions that had subtyping results, 408 (64.8%) were RSV-A, 204 (32.4%) were RSV-B, and 18 (2.9%) were both RSV-A and RSV-B positive.

## Discussion

Since expiration of the COVID-19 pandemic public health emergency declaration in May 2023 (*2*), CDC has continued efforts to optimize and integrate reporting and communication of national surveillance data for respiratory viruses. This report analyzed data from six complementary national surveillance systems to summarize the 2024–25 season for most respiratory viruses of public health significance.

The bimodal periodicity of positive SARS-CoV-2 test results observed in past years (*3*) continued during the 2024–25 season, including a larger July peak followed by a smaller January peak. Consistent with recent years preceding the 2024–25 respiratory virus season, COVID-19 hospitalization rates were highest among adults aged ≥75 years, followed by infants aged <6 months who are not eligible for COVID-19 vaccination, and adults aged 65–74 years. Maternal COVID-19 vaccination is the only available immunization strategy to protect infants aged <6 months from severe COVID-19. During 2024–2025, no major SARS-CoV-2 strain replacement occurred; all sequenced SARS-CoV-2 viruses circulating in the United States remained descendants of JN.1. The 2025–26 COVID-19 vaccines are expected to provide protection against severe disease (*4*–*6*), including from the XFG variant that approached predominance in late June 2025.**

During 2024–2025, RSV activity followed typical seasonal patterns observed before the COVID-19 pandemic (i.e., increased activity during October–April) that aligned with the timing of RSV vaccination guidance for adults and children for most of the United States. An interim evaluation based on 2024–2025 NVSN and RSV-NET data found that after the introduction of pediatric RSV prevention products, RSV-associated hospitalization rates in infants aged 0–7 months were reduced 28%–43%, compared with seasons preceding the introduction of those products (*7*). However, during 2024–2025, infants aged 0–11 months still experienced the highest RSV-associated hospitalization rates among all age groups, followed by children aged 12–23 months and adults aged ≥75 years. Three options for protecting infants in their first RSV season against severe RSV disease are available: a maternal RSV vaccine given during pregnancy at 32–36 weeks’ gestation and one of two long-acting monoclonal antibodies (nirsevimab or clesrovimab) administered to infants aged <8 months who are born during or entering their first RSV season (*8*). A single dose of RSV vaccine is recommended for all adults aged ≥75 years and for adults aged 50–74 years at increased risk for severe RSV disease (*9*).

### Limitations

The findings in this report are subject to at least four limitations. First, NREVSS is a passive and voluntary surveillance system; therefore, data from participating laboratories vary by season and might not be representative of all geographic areas or age groups. Second, because hospitalization rate estimates from NVSN and RSV-NET are generated using different methods and surveillance catchment areas, variations in point estimates, as noted for COVID-19 and RSV hospitalization estimates, are expected. Third, continuing declines in submitted SARS-CoV-2 specimens and resources available for sequencing over time affect the precision of genomic surveillance estimates. Finally, death certificate data likely do not include all deaths from COVID-19 and RSV because not all persons who die after COVID-19 or RSV are tested for these viruses, and some deaths might be attributed to associated complications, such as myocardial infarction or pneumonia. Therefore, deaths from NVSS should be considered minimum counts, whereas CDC estimates of COVID-19 and RSV mortality, which use continuously updated surveillance data, data from the latest scientific reports, and mathematical modeling, account for incomplete data. Because death certificates contain limited information on missed opportunities for intervention and potential risk factors for death, standardized national surveillance and reporting for pediatric laboratory-confirmed COVID-19 and RSV-associated deaths is being implemented in 2026.

### Implications for Public Health Practice

Core prevention strategies to reduce the risk for respiratory virus infections include staying up to date with vaccinations, washing hands regularly and cleaning commonly touched surfaces, optimizing ventilation in places where persons live and work, and staying home and away from others when sick. Available antiviral treatments for COVID-19 and influenza are underused (*10*), despite evidence that they decrease risk for hospitalization and death; early treatment might help decrease the risk for severe illness. CDC continues to monitor respiratory virus activity in the United States and provide weekly updates online.

## Footnotes

* 45 C.F.R. part 46, 21 C.F.R. part 56; 42 U.S.C. Sect. 241(d); 5 U.S.C. Sect. 552a; 44 U.S.C. Sect. 3501 et seq.

† NVSN test positivity data for children and adolescents aged <18 years are also available online. Pediatric Acute Respiratory Illness (ARI) Interactive Dashboard | NVSN | CDC

§ RSV season onset and offset were identified, respectively, as the week ending date corresponding to the first and last of 2 consecutive weeks when the percentage of weekly tests positive for RSV was ≥3%.

¶ For assessment of RSV, HHS Region 4 excludes Florida, HHS Region 9 excludes Hawaii, and HHS Region 10 excludes Alaska to account for notable variations in seasonal activity patterns in these states.

** 2025–2026 COVID-19 vaccines in the United States are JN.1 lineage-based with a preference for an LP.8.1-like antigen.
